# Feasibility study of the Four-Session Cognitive-Behavioral Therapy–based Psychological Intervention Program for women with HER2-positive metastatic breast cancer

**DOI:** 10.1007/s00520-026-10782-z

**Published:** 2026-05-16

**Authors:** Tamás Szekeres, Máté Ádám Balázs, Bálint Madarász, Zsuzsa Póti, Róbert Urbán, Magdolna Dank, Gabriella Vizin

**Affiliations:** 1 Faculty of General Medicine, Department of Internal Medicine and Oncology, Oncology Profile, Semmelweis University, Budapest, Hungary; 2 Institute of Psychology, Eötvös Loránd University, Budapest, Hungary; 3 National Institute of Oncology, Budapest, Hungary; 4 Oncoradiology Department, Uzsoki Street Hospital, Budapest, Hungary; 5 Department of Psychology, College of Health, Atlántico Medio University, Las Palmas de Gran Canaria, Spain

**Keywords:** HER2-positive breast cancer, Low-intensity psychological intervention, Palliative care, Psycho-oncology, Cognitive-behavioral therapy, Distress

## Abstract

**Purpose:**

Although targeted therapies have prolonged survival in human epidermal growth factor receptor 2 (HER2)–positive metastatic breast cancer, the resulting chronic disease trajectory is frequently accompanied by substantial psychological burden. This study aimed to conduct a preliminary investigation into the feasibility and psychological outcomes of a low-intensity, group-based cognitive-behavioral therapy intervention (CBT-OP-4) among women receiving palliative care.

**Methods:**

A prospective feasibility study without a control group was undertaken involving 13 female participants (mean age = 63 years, SD = 8.05), all diagnosed with HER2-positive metastatic breast cancer. The four-session CBT-based group intervention targeted key psychological domains. Self-reported measures of distress, depression, anxiety, perceived stigma, self-compassion, and locus of control were administered pre- and post-intervention. Feasibility and acceptability were further examined via semi-structured qualitative interviews and standardized patient satisfaction instruments.

**Results:**

Following the intervention, pre–post improvements were observed in psychological distress, depressive symptoms, and perceived stigma. In parallel, participants demonstrated an increase in self-compassion. Overall, the program was rated as highly acceptable, with participants emphasizing the relevance and applicability of the intervention content within their illness context.

**Conclusion:**

Findings from this preliminary study suggest that the CBT-OP-4 program is a feasible and acceptable psycho-oncological intervention for women with advanced-stage HER2-positive breast cancer receiving palliative care. The results offer initial empirical support for the incorporation of structured, evidence-based psychological interventions into the routine psychosocial management of this patient population.

## Introduction

Breast cancer remains the most commonly diagnosed malignancy among women worldwide [1]. The classification of breast cancer subtypes—such as luminal A, luminal B, human epidermal growth factor receptor 2 (HER2)-positive, and basal-like—is essential for developing personalized treatment strategies, as each subtype is associated with distinct prognoses and therapeutic responses [2]. The HER2-positive subtype accounts for approximately 15–20% of cases and is characterized by aggressive clinical behavior, rapid progression, and a higher recurrence rate [3]. Although HER2-targeted therapies (e.g., trastuzumab, pertuzumab) have significantly improved patient outcomes, in metastatic settings the disease remains a chronic, incurable condition that requires complex treatment and a multidisciplinary care approach [4].

Despite the survival benefits achieved with anti-HER2-targeted therapies in patients with metastatic disease, the psychosocial burden during the chronic, non-curative phase remains substantial [5]. These patients typically undergo continuous maintenance therapy, resulting in ongoing exposure to treatment-related side effects such as cardiotoxicity, altered immune response, gastrointestinal disturbances, and dermatological or hair-related issues [6]. Moreover, research has shown that a diagnosis of metastatic disease is frequently associated with elevated levels of psychological distress, depressive and anxiety symptoms, and perceived stigma [6, 7]. These psychological burdens are associated with reduced quality of life, may compromise treatment adherence, and have been linked to poorer overall survival [8].

In palliative care, there is an increasingly recognized need for integrated psychological interventions, a demand supported by the growing body of meta-analytic evidence demonstrating their positive effects on psychological outcomes [9]. Evidence-based psychotherapeutic approaches such as cognitive-behavioral therapy (CBT), mindfulness-based stress reduction (MBSR), self-compassion training, and positive psychology interventions have shown effectiveness in reducing psychological distress, depression, anxiety, and perceived stigma. These approaches also contribute to improved quality of life, enhanced coping skills, and, in some cases, better adherence and survival outcomes [10–14].

Although comprehensive CBT-based programs have been developed to improve quality of life and treatment adherence among oncology patients [15], no such integrated program has been specifically tailored to the psychological needs of patients with HER2-positive metastatic breast cancer receiving palliative care (e.g., trastuzumab, pertuzumab). To date, there has been no comprehensive intervention that incorporates evidence-based psychotherapeutic tools adapted to the complex psychosocial context of this patient population.

Although a growing body of literature supports the effectiveness of psychological interventions in oncology, relatively few studies have examined brief or low-intensity interventions specifically in palliative care or metastatic breast cancer populations. Existing programs are often longer in duration or require substantial patient engagement, which may limit their feasibility in populations characterized by advanced disease, ongoing treatment burden, and reduced physical and psychological capacity. Consequently, there is a need for shorter, accessible, and clinically relevant interventions tailored to the specific needs of patients receiving palliative care. The present study addresses this gap by evaluating the feasibility and preliminary psychological outcomes of a brief, four-session CBT-based group intervention (CBT-OP-4) in women with HER2-positive metastatic breast cancer.

The aims of the present study are (1) to provide a brief overview of a comprehensive, low-intensity, CBT-based group intervention (Four-Session Cognitive-Behavioral Therapy–based Psychological Intervention Program for Oncological Patients; CBT-OP-4), specifically developed for women with HER2-positive metastatic breast cancer receiving palliative care; and (2) to assess the feasibility and report preliminary psychological outcomes associated with the intervention.

## Methods

### Sample and procedure

The present study represents a prospective feasibility sub-study embedded within a larger investigator-initiated study (IIS), focusing specifically on the implementation and preliminary evaluation of a CBT-based group intervention (CBT-OP-4).

**Fig. 1:**
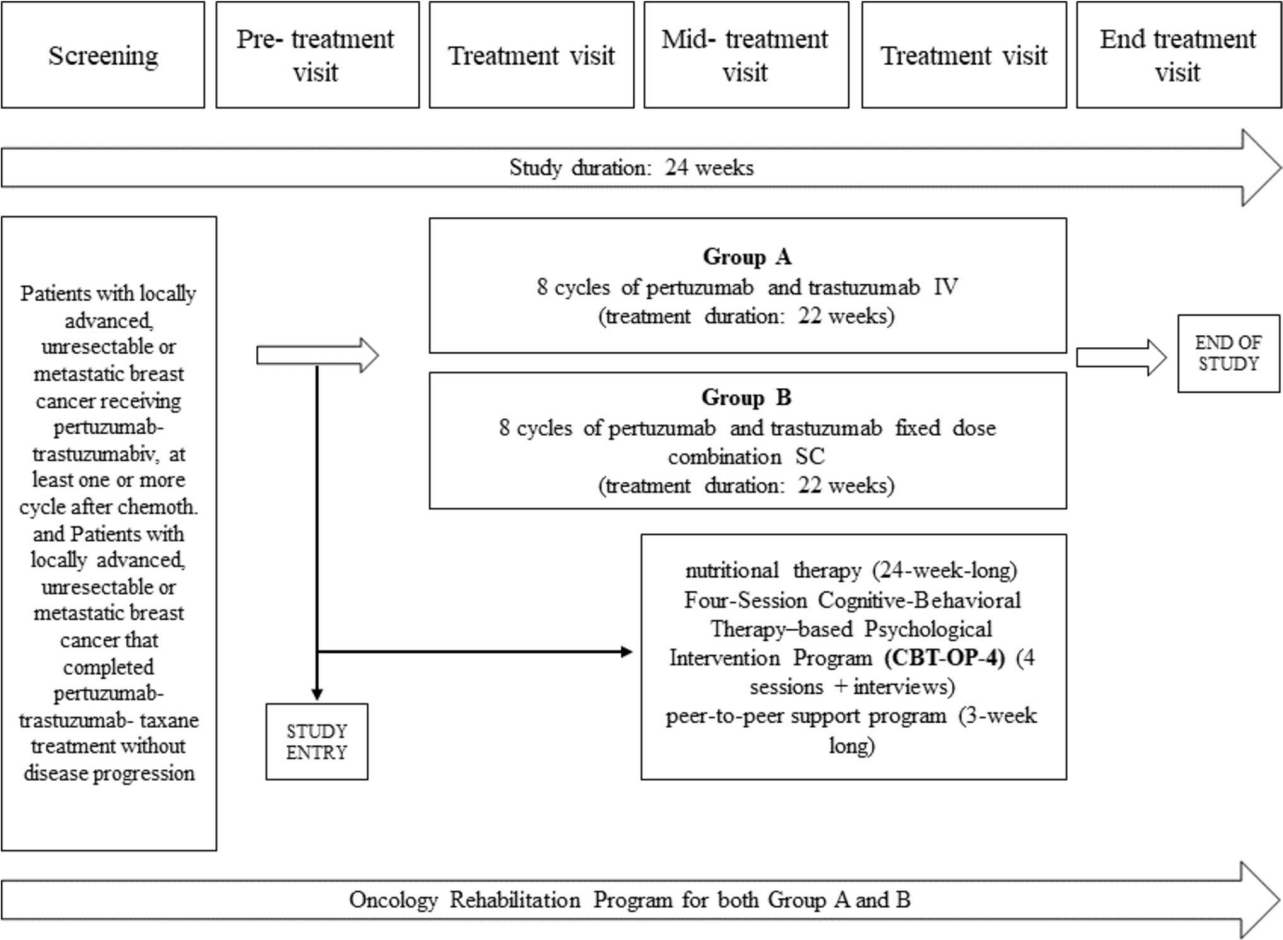
Study design

The prospective feasibility study was conducted within an investigator-initiated academic study (IIS)—titled “Value-based health economic comparative study of subcutaneous and intravenous pertuzumab-trastuzumab for HER2 positive metastatic breast cancer based on patient reported outcomes.”—in 2023–2025. The overall study aimed to perform a value-based health economic comparison of subcutaneous and intravenous pertuzumab-trastuzumab for HER2-positive metastatic breast cancer based on patient-reported outcomes. In addition, the IIS included a comprehensive onco-rehabilitation program, comprising peer support, nutritional therapy, and a brief CBT-based psychological intervention (CBT-OP-4). The full study protocol is illustrated in Fig. 1.

Participants were recruited from multiple oncology centers in Hungary. A total of 23 patients participated in the study, including 16 from the Oncology Profile of the Department of Internal Medicine and Oncology at Semmelweis University, 5 from Uzsoki Street Hospital, 1 from the University of Debrecen, and 1 from the South-Pest Central Hospital.

The intervention group (*n* = 13) included participants from the University of Debrecen (*n* = 1), Uzsoki Street Hospital (*n* = 2), and Semmelweis University (*n* = 10).

**Fig. 2:**
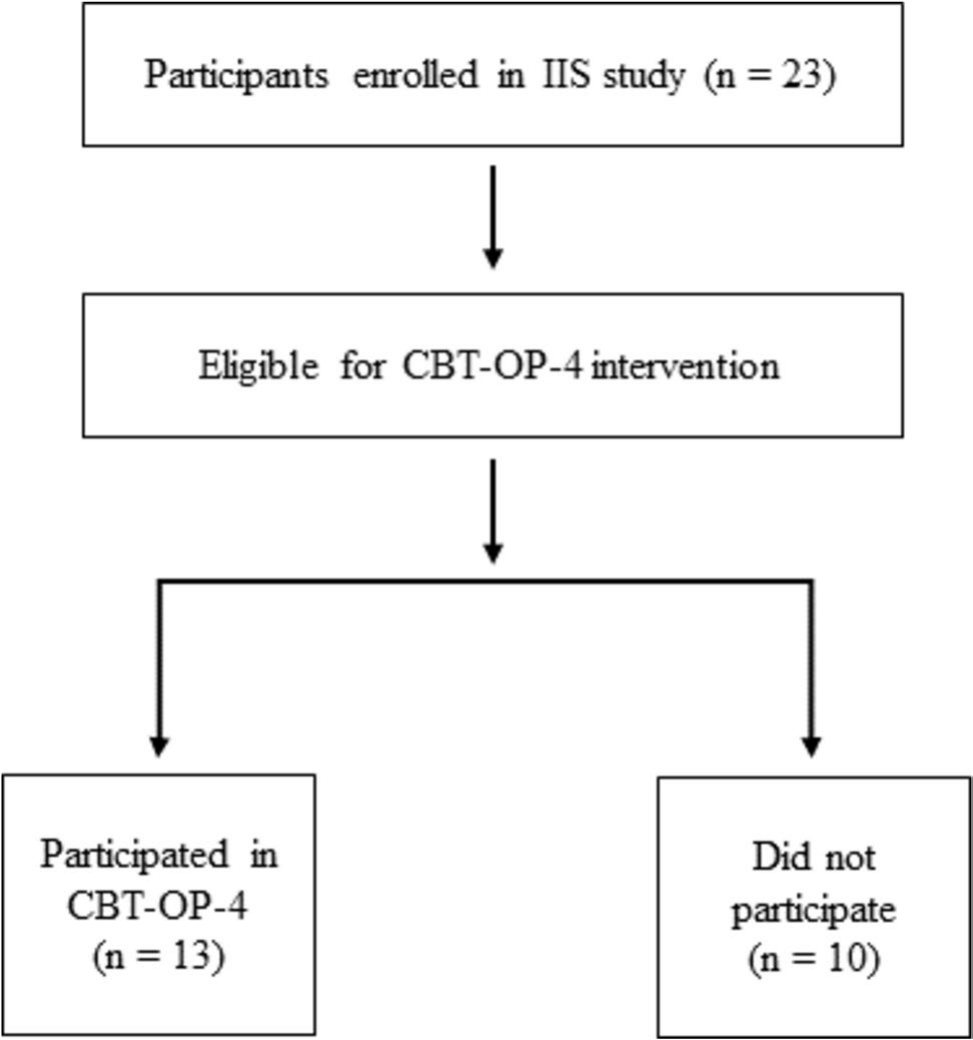
Participant flow of the CBT-OP-4 feasibility sub-study within the IIS cohort. Note. The diagram illustrates the selection of participants from the overall IIS sample (*n* = 23) into the CBT-OP-4 intervention group (*n* = 13) and the non-participating group (*n* = 10), based on eligibility, willingness, and availability

Participation in the CBT-OP-4 intervention was not randomized. All patients enrolled in the IIS study who met the inclusion criteria for the psychological program were informed about the opportunity to participate in the group-based intervention. Participation was voluntary and based on patients’ willingness and availability to attend the scheduled group sessions. Due to logistical constraints (e.g., group size limits and scheduling), a subset of the total IIS sample (*n* = 13) took part in the CBT-OP-4 program, while the remaining participants did not receive the psychological intervention. No systematic allocation procedure was applied. No formal control group was defined for the present feasibility sub-study; participants who did not take part in the intervention served as a non-intervention comparison group within the broader IIS cohort. The participant flow and allocation are illustrated in Fig. 2.

The study sample consisted of patients enrolled in the IIS; sampling was voluntary and based on strict inclusion and exclusion criteria.

The inclusion and exclusion criteria of the overall study also applied to the CBT-OP-4 psychological support program. The following inclusion criteria were required: written informed consent from the patient; the patient had to finish induction therapy with pertuzumab and trastuzumab with chemotherapy for breast cancer; age of at least 18 years at the time of enrollment; confirmed HER2-positive, locally advanced, unresectable or metastatic breast cancer, confirmed by imaging and histopathological examination, irrespective of hormone receptor status; ability to comply with the study protocol; Eastern Cooperative Oncology Group (ECOG) performance status of 0–1; good patient adherence; baseline left ventricular ejection fraction (LVEF) of at least 50% measured by echocardiography; no contraindications of pertuzumab and trastuzumab treatment; and an expected survival exceeding six months.

Patients were excluded from the study if they met any of the following exclusion criteria: use of a port catheter for intravenous drug administration; age above 75 years; early-stage breast cancer without confirmed local or distant metastases; progression during pertuzumab and trastuzumab therapy; severe cardiovascular comorbidities, especially New York Heart Association (NYHA) functional class II–IV; pregnancy or lactation; presence of severe psychiatric disorders; current severe, uncontrolled systemic disease that may interfere with planned treatment (e.g., clinically significant cardiovascular, pulmonary, or metabolic disease; wound-healing disorders); inadequate bone marrow function; impaired liver function; and impaired renal function (serum creatinine > 1.5 × upper limit of normal). Any medical condition or laboratory abnormality that, in the investigator’s judgment, would jeopardize the patient’s safe participation in the study also led to exclusion. Additionally, patients with active liver disease (e.g., hepatitis B or C infection, autoimmune hepatitis, sclerosing cholangitis), concurrent severe uncontrolled infections, known HIV infection, or known hypersensitivity to the investigational drugs, excipients, or murine-derived proteins, were excluded. Table 1 presents the descriptive statistics of the study sample.

**Table 1:** Descriptive characteristics of the CBT-OP-4 intervention sample (feasibility sub-study)

Variable	Frequency/mean (SD)
**Age**	63 (8.05)
**Relationship status** Widowed Divorced Married In a relationship	3 (23%) 2 (15%) 6 (47%) 2 (15%)
**Place of residence** Capital city Village Town/city	5 (38%) 2 (15%) 6 (47%)
**Educational attainment** Elementary school Vocational school/technical school High school Bachelor’s degree (BSc) Master’s degree (MSc)	1 (8%) 1 (8%) 3 (23%) 7 (53%) 1 (8%)
**Number of years since diagnosis**	10.61 (6.87)
**Elapsed time (in years) since the diagnosis of metastatic disease**	7.38 (5.59)
**Hormone receptor status** Estrogen receptor (ER) positive Hormone receptor negative	4 (31%) 9 (69%)
**Site(s) of metastasis** Skin Bone Bone and lymph nodes Bone and liver Liver and lung Lymph nodes and brain Lymph nodes Lung	1 (8%) 3 (23%) 2 (15%) 2 (15%) 1 (8%) 1 (8%) 2 (15%) 1 (8%)

No additional inclusion or exclusion criteria were applied specifically for participation in the CBT-OP-4 intervention beyond those defined for the broader IIS study. Participation in the psychological intervention was based on eligibility within the IIS study, as well as patients’ willingness and availability. Given the exploratory nature of this feasibility study, no formal sample size calculation was performed. The sample size was determined pragmatically based on available participants within the IIS study.

Due to publication sequencing, the present analysis of psychological outcomes is reported prior to the publication of the primary trial endpoints. The psychological outcomes are independent of the primary endpoint and their reporting does not affect the integrity of the main trial results.

The manuscript was reviewed in light of the CONSORT Extension for Pilot and Feasibility Trials, and reporting was aligned with its recommendations where applicable.

The study has been registered in the European Medicines Agency (EMA) clinical trials database (EudraCT No. 2023-000156−38), registered on May 2, 2023. The study protocol number is 20230123SE. The study was approved by the Hungarian Health Authority.

### Ethics

The study was conducted in accordance with the latest version of the Declaration of Helsinki and complied with applicable Good Clinical Practice (GCP) guidelines. Ethical approval for the research was granted by the Medical Research Council – Ethics Committee for Clinical Pharmacology (MRC ECCP - ETT KFEB), under the reference number BM/6420–0/2023-EKL.

Consent to participate: All participants provided informed consent prior to inclusion in the study.

### Interventions (Four-Session Cognitive-Behavioral Therapy–based Psychological Intervention Program for Oncological Patients, CBT-OP-4)

Participants took part in a 4-week, low-intensity, group-based cognitive-behavioral therapy intervention consisting of four sessions. Each session lasted 120 min and was facilitated by a cognitive-behavioral therapy (CBT)–trained therapist alongside a clinical psychologist with specialized training in CBT.

The intervention was based on the CBT-based psychological intervention program for Oncological Patients (CBT-OP), developed by Vizin [16]. This program was originally designed to reduce psychological symptoms related to cancer and its treatments, and to enhance treatment adherence, specifically for women with grades I–III breast cancer. The original program consists of ten group sessions, with core components including cognitive restructuring, behavioral activation, problem-solving, and education and psychoeducation, as well as mindfulness and self-compassion meditation. We adapted this program for use with women receiving palliative care.

At the beginning of the program, participants received a workbook containing diaries, psychoeducational materials, and related worksheets. Each session commenced with a review of the home practice diaries. Every session included psychoeducational components, psychological intervention elements, home practice assignments, and group discussions related to these activities. Participants were sent reminder emails on the day following each session, which included materials for the week’s exercises. Sessions concluded with brief mindfulness and self-compassion meditation practices. The CBT-OP-4 program was structured as follows (Table 2):

**Table 2:** Structure of the Four-Session Cognitive-Behavioral Therapy–based Psychological Intervention Program for Oncological Patients (CBT-OP-4)

Session	Topic	Intervention
*Psychological assessment*	Assessed domains: depression, anxiety, distress, problem checklist, levels of stigmatization and shame, and self-compassion	Distress Thermometer, PHQ-4, SSCI-8, MHLC-C, SCS-SF
*First session*	Psychoeducation, emotion validation, cognitive model	cognitive conceptualization, Three-column reflective journal
*Second session*	Depression and anxiety education, behavioral activation	List of pleasant activities, mood diary
*Third session*	Assertiveness, resilience	Assertiveness training, resilience exercise [34], gratitude journal
*Fourth session*	Crisis, coping, problem-solving	Psychoeducation on the cognitive model of coping with cancer, problem-solving worksheet
Mindfulness and self-compassion practice at the end of each session		
*Psychological assessment*	Assessed domains: depression, anxiety, distress and problem checklist, levels of stigmatization and shame, self-compassion	Distress Thermometer, PHQ-4, SSCI-8, MHLC-C, SCS-SF
*Feasibility*	Evaluation of the program’s effectiveness and relevance	Semi-structured interview

*PHQ-4*, four-item patient health questionnaire; *MHLC*, Multidimensional Health Locus of Control Scales; *SCS-SF*, Self-Compassion Scale; *SSCI-8*, Stigma Scale for Chronic Illnesses questionnaire

The CBT-OP-4 program was developed as a condensed adaptation of the original 10-session CBT-OP protocol. Core therapeutic components of the original intervention—such as psychoeducation, cognitive restructuring, behavioral activation, problem-solving, and elements of mindfulness and self-compassion—were retained. Due to the reduced number of sessions, certain elements were simplified and integrated into combined sessions rather than being delivered as standalone modules. The adaptation process prioritized clinically relevant skills that could be effectively applied in a palliative care context, with an emphasis on emotional regulation, coping with illness-related distress, and enhancing self-compassion. The rationale for the shortened, four-session format was to reduce participant burden and improve feasibility in a population characterized by advanced disease status, ongoing treatment, and limited physical and psychological capacity. The structure was therefore designed to balance therapeutic depth with accessibility and practical applicability in a real-world clinical setting.

Participants’ psychological status was assessed at two time points: prior to the intervention (baseline) and post-intervention. Validated self-administered questionnaires were employed to measure the psychological effects.

### Measures

*Demographic and disease-related data*: Data collection on demographic and disease-specific variables included age, relationship status, educational attainment, type of residence, date of initial breast cancer diagnosis, date of metastatic breast cancer diagnosis, hormone receptor status, and sites of metastasis.

*Distress Thermometer *[17]: To measure psychological distress, the Distress Thermometer was employed, which is a single-item, 11-point visual analog scale ranging from 0 to 10. A score of 0 indicates no distress, while 10 represents the highest level of distress. Participants rated the level of distress they experienced over the past week, including the current day, by selecting a number on the scale. Complementing the scale is a 40-item problem checklist designed to identify sources of distress across six thematic categories: practical, family, emotional, spiritual/religious, physical, and other problems. Respondents indicated “yes” or “no” for each item depending on whether they experienced the specific problem during the past week.

*Four-item patient health questionnaire for anxiety and depression (PHQ-4) *[18]: The PHQ-4 is an ultrabrief, four-item self-administered psychological screening tool designed to rapidly detect symptoms of depression and anxiety. The questionnaire combines the Generalized Anxiety Disorder-2 (GAD-2) and the Patient Health Questionnaire-2 (PHQ-2), each comprising two items that measure symptoms of anxiety and depression, respectively [18]. Responses are given on a 4-point Likert scale (0–3), reflecting symptom frequency over the past 2 weeks. The total score ranges from 0 to 12 and indicates the overall level of psychological distress, while subscale scores for anxiety and depression can be evaluated separately. The PHQ-4 demonstrates excellent psychometric properties and has been validated across various clinical and non-clinical populations, including oncology patients. The scale exhibits high sensitivity and specificity, making it particularly suitable for low-intensity settings and somatically burdened, older populations.

*Stigma Scale for Chronic Illnesses (SSCI-8) questionnaire *[19, 20]: To assess stigmatization, the 8-item Stigma Scale for Chronic Illnesses (SSCI-8) was utilized. The questionnaire consists of eight items, making it suitable for use in clinical settings [20]. Participants rate statements such as “I felt embarrassed about my illness” and “Some people treated me as if it was my fault that I have this illness” on a 4-point Likert scale (1 = never, 4 = always). Scores range from 8 to 40, with higher scores indicating greater perceived stigmatization [20]. The instrument has demonstrated validity and reliability, with a Cronbach’s alpha of 0.89 [19, 21]. Among samples of women with breast cancer, it has shown good test–retest reliability (*r* = 0.78) and internal consistency (Cronbach’s alpha = 0.89) [22].

*Form C of the Multidimensional Health Locus of Control Scales (MHLC-C) questionnaire *[23, 24]: To assess health-related locus of control, the Hungarian version of the Multidimensional Health Locus of Control (MHLC) Form C questionnaire was utilized [ 25]. This instrument is specifically designed to evaluate the health locus of control among individuals living with chronic illnesses. The questionnaire comprises three subscales: internal control, external control (chance or luck), and social control. It contains no reverse-scored items. Individuals perceiving a high level of internal control believe they actively influence their own health. When the “chance” factor predominates, the course of the illness appears more unpredictable to the respondent. External control is characterized by a belief that the outcome of the illness is primarily determined by physicians or other significant individuals whom the person trusts.

Self-compassion was assessed using the Hungarian adaptation of the 12-item short form of the *Self-Compassion Scale (SCS-SF)* [26, 35]. The short form has demonstrated good reliability and validity and is suitable for assessing overall self-compassion in research settings. The scale consists of 12 items rated on a five-point Likert scale ranging from “almost never” to “almost always.” After reversing negatively worded items, scores were summed to calculate an overall self-compassion score, with higher scores indicating greater self-compassion [26]. Previous studies have demonstrated good psychometric properties for the short form, with acceptable to good internal consistency (Cronbach’s alpha = 0.86) [27, 35].

*Semi-structured interviews on feasibility*: To gain a deeper understanding of the program’s effectiveness and relevance, semi-structured interviews were conducted with participants of the intervention. The interviews were carried out by a researcher with clinical psychologist qualifications. The purpose of the interviews was to explore participants’ subjective experiences regarding the usefulness of the group sessions, as well as the extent to which the intervention techniques learned could be integrated into their daily lives. During the interviews, participants were asked questions such as “Do you think the program addresses problems common among individuals with breast cancer?” and “Do you believe the skills taught in the program would be relevant for others in similar situations to yours?” Additionally, participants evaluated how easy they found the application of the learned techniques in practice (“I thought the learned techniques were easy to use”). The practical utility and applicability of each intervention element were also assessed using a 10-point Visual Analog Scale (VAS), allowing for the quantitative recording of experiences. Responses from the semi-structured interviews were analyzed descriptively, with key themes summarized to reflect participants’ experiences and perceptions of the intervention. No formal qualitative coding or thematic analysis procedure was applied.

### Statistical processing

IBM SPSS 23.0© and JASP software were used for data analyses. First, the internal consistency of the measurement instruments employed in the study was assessed by calculating Cronbach’s alpha coefficients, all of which met acceptable reliability standards. Second, exploratory pre–post comparative analyses were conducted to examine potential within-group changes associated with the intervention. Effect sizes were estimated using Hedges’ *g*, which corrects for small sample bias [28]. Effect size magnitudes were interpreted using conventional benchmarks (0.20 = small, 0.50 = medium, 0.80 = large), commonly applied to standardized mean differences [27]. However, in psycho-oncological and palliative care contexts, even small effect sizes may be clinically meaningful, particularly regarding quality of life and psychological well-being. The 95% confidence intervals for Hedges’* g* were calculated using its standard error and an appropriate critical *t*-value to account for small sample variability [29]. Finally, qualitative data collected through semi-structured interviews regarding the program interventions are presented, focusing on the current sample.

## Results

Feasibility outcomes are presented first, followed by exploratory analyses of pre–post psychological changes.

### Feasibility outcomes

Feasibility indicators were assessed in terms of recruitment, retention, and session attendance. Of the total IIS sample, 13 participants were enrolled in the CBT-OP-4 intervention. No participants dropped out after initiating the program, resulting in a retention rate of 100%. Session attendance was exceptionally high: only one participant missed a single session, corresponding to an overall attendance rate of 98.1%. These findings indicate good feasibility and acceptability of the intervention in this patient population. A summary of feasibility indicators is presented in Table 3.

**Table 3:** Feasibility indicators of the CBT-OP-4 intervention

Indicator	Value
IIS sample (*n*)	23
Participated in CBT-OP-4 (*n*)	13
Recruitment rate (%)	56.5%
Dropout (*n*, %)	0 (0%)
Attendance rate (%)	98.1%

Feasibility indicators include recruitment, retention, and session attendance rates among participants enrolled in the CBT-OP-4 program

### Patient satisfaction

To assess the perceived effectiveness and relevance of the program, semi-structured interviews were conducted with participants to evaluate patient satisfaction. Based on the responses, the psychotherapeutic group received highly positive feedback from women living with metastatic breast cancer. The overall usefulness of the group was rated with an average score of 9.69 (SD = 0.63) on a 10-point scale, while the therapeutic applicability of the learned techniques during treatment received a mean score of 9.54 (SD = 0.66). In response to a general question regarding the frequency of applying the learned techniques in daily life, 8% of participants reported using the techniques weekly, 77% used them multiple times per week, and 15% applied them daily. These responses refer to overall use of the techniques. In contrast, Table 4 presents frequency ratings for individual intervention components, which were assessed separately.

**Table 4:** Descriptive analysis of patient satisfaction outcomes pertaining to the implemented interventions

Topic	Questions	Mean (SD)/frequency Min, max
General questions	How useful did you find the group sessions?	9.69 (0.63) Min 8 Max 10
	To what extent do you feel able to apply the skills learned during the group sessions throughout your treatment?	9.54 (0.66) Min 8 Max 10
	How frequently have you utilized the techniques learned in the group sessions in your daily life?	8% reported using the techniques weekly, 77% used them multiple times per week, and 15% applied them daily
Disease-specific fit	Do you believe that the program addresses issues commonly experienced by individuals living with breast cancer?	9.76 (0.44) Min 9 Max 10
	Do you think that the skills taught in the program would be relevant to other people in similar situations to yourself?	Yes 100%
Assessment of interventions	I found the learned techniques easy to use	Yes 77% No 23%
	Mindfulness practice	9.30 (0.63) Min 8 Max 10
	Mood diary	9.53 (0.52) Min 9 Max 10
	Self-compassion meditation	9.61 (0.65) Min 8 Max 10
	List of pleasant activities/behavioral activation	8.61 (0.87) Min 7 Max 10
	Gratitude journal	9.46 (0.66) Min 8 Max 10
	Assertive communication	10 Min 10 Max 10
	Problem-solving worksheet	8.53 (0.87) Min 7 Max 10
Feasibility	Did you find the program sufficient?	Yes 100%
	Did the program adapt to your current life situation and difficulties?	Yes 100%
	Would you recommend participation in the group to individuals living with cancer?	10 Min 10 Max 10

The thematic relevance and alignment of the program were also rated highly (M = 9.76; SD = 0.44), and all participants agreed that the techniques taught could be beneficial for others in similar situations.

The individual components of the intervention were also evaluated positively. Mindfulness exercises were rated as useful by 77% of respondents, self-compassion meditations by 100%, and gratitude and mood journaling practices by 92%. Behavioral activation, assertive communication techniques, and the problem-solving worksheet also received favorable ratings, each with an average score above 8.5.

Regarding feasibility, 100% of participants reported that the group was well adapted to their current life circumstances and challenges. All respondents indicated that they would highly recommend the program to others living with cancer, giving it a maximum score (10/10). The results are summarized in Table 4.

### Pre- and post-intervention results

Exploratory pre–post analyses were conducted to examine potential changes in psychological outcomes within the intervention group. Table 5 presents the descriptive statistics of the applied questionnaires, separated by pre- and post-intervention assessments. The results indicate a statistically significant medium effect size difference in the level of depression, distress, family problems, social control, self-compassion, and stigmatization. In other words, pre–post comparisons within the intervention group indicated lower levels of distress, fewer interpersonal conflicts, reduced depressive symptoms, and decreased stigmatization. Moreover, participants reported a reduced perception of the influence of significant others on their illness outcome, while their self-compassion increased.

**Table 5:** Descriptive and inferential statistics for pre- and post-intervention assessment

Variable	Pre		Post		*t*	*p*	r*	Hedges’ *g* [95% CI]
	Mean	SD	Mean	SD				
Distress thermometer	4.15	2.76	2.00	2.44	2.569	**0.025**	0.33	0.667 [0.082, 1.229]
Practical problems	1.15	1.21	1.30	1.31	−0.395	0.700	0.38	−0.045 [−0.496, 0.408]
Family-related issues	0.94	0.49	0.46	0.66	2.144	**0.050**	0.12	0.557 [0.008, 1.102]
Emotional difficulties	2.23	1.78	1.69	1.31	1.460	0.170	0.67	0.332 [−0.138, 0.793]
Physical symptoms	5.46	2.50	5.53	2.96	−0.143	0.888	0.76	0.000 [−0.452, 0.452]
Depression (PHQ-4)	3.34	2.80	2.06	1.31	2.310	**0.034**	0.56	0.532 [0.040, 1.011]
Locus of control (MHLC)								
External control	14.53	7.45	11.53	4.85	1.811	0.095	0.60	0.453 [−0.030, 0.923]
Internal control	21.46	5.98	19.23	8.32	1.049	0.315	0.77	0.196 [−0.264, 0.649]
Social control	25.46	4.09	23.69	4.13	2.379	**0.035**	0.78	0.616 [0.043, 1.172]
Self-compassion (SCS-SF)	96.07	4.23	98.84	5.09	−2.481	**0.029**	0.64	0.644 [0.065, 1.203]
Stigma (SSCI-8)	15.84	8.47	12.15	3.87	2.153	**0.050**	0.74	0.695 [0.177, 1.195]

*PHQ-4*, four-item patient health questionnaire; *MHLC,* Multidimensional Health Locus of Control Scales; *SCS-SF*, Self-Compassion Scale; *SSCI-8*, Stigma Scale for Chronic Illnesses questionnaire*.* Effect sizes are reported as Hedges’ *g* for paired (dependent) samples, corrected for small sample bias. *The value of *r* denotes the correlation between pre-test and post-test scores. This value is necessary for the computation of the effect size for a paired-samples (dependent samples) *t*-test. All *t*-tests were conducted as two-tailed tests. Bold values indicate statsitically significant results (p<0.05)

## Discussion

Importantly, this study was not designed to evaluate efficacy, and the observed changes should be considered preliminary and exploratory. The primary contribution of this study lies in demonstrating the feasibility and acceptability of the intervention, while the observed psychological changes provide initial signals that warrant further investigation in controlled trials.

This study is the first to examine the applicability of the Four-Session Cognitive-Behavioral Therapy-based Psychological Intervention Program for Oncological Patients (CBT-OP-4) low-intensity, structured psychological intervention in the palliative care of women living with HER2-positive metastatic breast cancer. Our findings suggest that the program is feasible within a therapeutic setting and may be associated with positive psychological changes, as indicated by both participants’ subjective evaluations and validated psychological assessment tools. Pre–post comparisons indicated that reductions were observed in levels of distress, depressive symptoms, family-related conflicts, and stigmatization, while improvements were noted in self-compassion and a reduced perception of the role of social control. However, results with *p*-values at the conventional threshold (e.g., *p* = 0.050) should be interpreted with caution. Given the small sample size, the exploratory nature of the analyses, and the absence of correction for multiple testing, such findings are best considered borderline and indicative of potential trends rather than definitive evidence. Accordingly, emphasis is placed on effect sizes and confidence intervals, and the results are framed as preliminary signals that warrant further investigation in larger, controlled studies.

These findings are consistent with previous studies demonstrating the effectiveness of cognitive-behavioral interventions in oncology populations [10, 11, 30]. Notably, these changes were observed after only four sessions, highlighting the potential feasibility of such a brief intervention format within the time constraints and capacity considerations of palliative care.

The systematic structure of the program—which integrates elements of psychoeducation, cognitive restructuring, behavioral activation, assertiveness, self-compassion, and coping—may have supported participants in processing the emotional and social challenges related to their illness in a differentiated manner.

According to semi-structured interviews and patient satisfaction feedback, participants perceived the skills acquired during the sessions as applicable in daily life, which may support their continued use. These findings underscore the potential practical relevance of psycho-oncological interventions even among patients with advanced-stage illness [15, 31].

Based on our findings, CBT-OP-4 appears to be a feasible component of psychological support interventions in palliative oncology care. The observed reduction in stigmatization and increase in self-compassion may be particularly relevant for patients whose illness is no longer curable and who often face vulnerability and social isolation [5, 32]. Improvements in self-compassion may serve as psychological resources supporting quality of life, although these effects should be interpreted cautiously given the uncontrolled design and small sample size [12, 26].

It is important to note that the program did not specifically target somatic symptoms or treatment-related side effects. While the observed patterns may be beneficial, any potential effects on overall well-being or treatment adherence remain hypothetical and should be interpreted with caution [9, 33]. Given the uncontrolled design, these findings should be interpreted as within-group observations, and alternative explanations (e.g., natural variation or external factors) cannot be excluded.

### Study limitations

Although the findings of this study are promising, several methodological and generalizability limitations must be considered. First, the small sample size considerably limits the statistical power and the validity of inferences. While the applied statistical tests and effect size estimations are indicative, the absence of a randomized controlled trial precludes drawing causal conclusions about the observed changes being solely attributable to the intervention. In addition, no correction for multiple testing was applied. Given the exploratory nature of the study and the small sample size, the results should therefore be interpreted with caution, particularly with respect to statistical significance.

Second, although the majority of patients enrolled in the study were recruited from a single institution, a few participants were included from other hospitals as well, which reduces the degree of homogeneity and slightly increases the generalizability of the findings.

Additionally, the socio-economic status distribution of the participants is not representative of the broader population of HER2-positive metastatic breast cancer patients.

Third, psychological effects were assessed using self-report instruments, which may be subject to social desirability bias. Although the structured interviews evaluating the program’s acceptability and feasibility provided valuable qualitative insights, they cannot replace efficacy assessments based on long-term follow-up studies.

Finally, although the CBT-OP-4 program is a structured, evidence-based psychological intervention, the intervention was not conducted in isolation but as part of a comprehensive oncological rehabilitation protocol that included peer-to-peer support, nutritional intervention, and medical monitoring, making it impossible to definitively isolate the effects of the individual therapeutic components.

### Clinical implications

Based on the findins of the present feasibility study, the CBT-OP-4 represents a feasible and potentionally clinically relevant low-intensity psychotherapeutic intervention that, according to our preliminary findings and patient satisfaction assessments, could be integrated into the psychosocial component of palliative care for patients with HER2-positive metastatic breast cancer. Pre-post changes were observed in distress, depressive symptoms, stigmatization, and self-compassion, which may reflect psychological processes relevant to adaptation to illness. Self-compassion, in particular, has been identified in previous research as a protective factor in psychological adaptation to illness [12, 26].

The obtained findings further support consideration of psycho-oncological interventions in palliative oncology care, particularly for patient groups who, despite disease progression, may experience longer survival due to targeted therapies [3]. Addressing the psychological burden of chronic illness may be important for supporting quality of life and treatment engagement [9, 10].

The structured nature, standardized interventions, thematic organization, and brief duration of the CBT-OP-4 protocol may facilitate broader implementation across various clinical settings. The group format may also allows for cost-effective and efficient delivery in health care environments where psychotherapeutic services are limited. This approach aligns with international efforts to strengthen the multidisciplinary and comprehensive perspective of palliative oncology care [31].

Clinically, the findings highlight the potential relevance of structured, targeted psychological interventions for patients with advanced breast cancer. However, given the exploratory and uncontrolled nature of the present study, any potential influence on treatment adherence or broader aspects of patient care should be interpreted cautiously and warrants further investigation [8, 11].

## Conclusion

The results of the present feasibility study suggest that the CBT-OP-4 program—as a structured, low-intensity, group-based psychological intervention—can be promisingly integrated into the palliative care of women living with HER2-positive metastatic breast cancer. Pre–post changes were observed in psychological distress, depressive symptoms, and stigmatization, alongside an increase in self-compassion, which may indicate psychological changes that could provide clinically relevant support for psychosocial adaptation to cancer diagnosis and treatment. The intervention’s favorable acceptability and high practical applicability further reinforce the justification for implementing the program within this particularly vulnerable patient population.

Although the small sample size and non-randomized design of the study limit the interpretation of the psychological outcomes, this research provides a valuable foundation for future longitudinal and controlled trials investigating the efficacy and long-term effects of the CBT-OP-4 protocol.

Our findings also support the necessity of the structured integration of psycho-oncological interventions into palliative care, particularly for patients facing significant psychosocial challenges despite extended survival due to targeted therapies.

## Data Availability

The datasets generated and/or analyzed during the current study are not publicly available due to the sensitive nature of the clinical and psychological data and the risk of participant re-identification, but are available from the corresponding author on reasonable request and with appropriate ethical approval.
